# When is a randomised controlled trial required: the theoretical domains framework approach

**DOI:** 10.1186/1745-6215-14-S1-O7

**Published:** 2013-11-29

**Authors:** Marion Campbell, Jill Francis, Eilidh Duncan, Graeme MacLennan, Brian Cuthbertson

**Affiliations:** 1 University of Aberdeen, Aberdeen, UK; 2 City University, London, UK; 3 Sunnybrook Health Sciences Centre, Toronto, Canada

## 

When evidence of a potentially promising intervention starts to accumulate it is often difficult to know whether the evidence is strong enough to move to promote widespread adoption or whether any, or further, randomised trials are required. We propose that the "Theoretical Domains Framework", or TDF, is a useful tool to guide whether further randomised trials require to be undertaken. The TDF is a theory-informed framework developed in the field of health psychology, that allows the systematic assessment of constructs likely to affect health professionals adoption/use of the intervention under consideration. It assesses twelve separate domains that may affect health professionals readiness to adopt a treatment or change their behaviour. Depending on the profile of responses to the TDF, decision rules can be generated to determine whether further effectiveness research is still required. We recently adopted the TDF approach in a critical care setting exploring whether further randomised trials of a particular treatment (selective decontamination of the digestive tract) were deemed to be required (and, if so, additional questions identified what particular aspects should be addressed). This mixed-methods international study involving research groups in the UK, Canada, Australia and New Zealand highlighted the usefulness of the TDF approach in providing an evidence-based judgement on whether a randomised trial should be initiated. We will explain the TDF approach, how it can be adopted to identify whether further trials are required, and demonstrate its use in practice.

